# Prevalence of obesity and diabetes in older people with sarcopenia defined according to EWGSOP2 and FNHI criteria

**DOI:** 10.1007/s40520-021-01949-1

**Published:** 2021-08-16

**Authors:** Francesca Remelli, Elisa Maietti, Pasquale Abete, Giuseppe Bellelli, Mario Bo, Antonio Cherubini, Francesco Corica, Mauro Di Bari, Marcello Maggio, Maria Rosaria Rizzo, Andrea P. Rossi, Francesco Landi, Stefano Volpato, the GLISTEN Group Investigators, Gloria Brombo, Beatrice Ortolani, Elisabetta Savino, Elisa Maietti, Alberto Fisichella, Valeria Buttò, Mauro Zamboni, Cesare Caliari, Elena Ferrari, Francesco Orso, Flavia Sacco, Maria Laura Di Meo, Anna Paola Cerri, Marco Motta, Francesca Pittella, Alessandra Bonfanti, Sergio Fusco, Roberto Schepisi, Christian Ferro, Antonino Catalano, Stefano Caruso, Luca Soraci, Lorenzo Marchese, Luca Agosta, Claudia Basile, Carla Coppola, Anna Maria Dalise, Ilaria Fava, Olga Catte, Maura Orru’, Paolo Salaris, Anna Maria Martone, Elena Ortolani, Sara Salini, Giuseppina dell’Aquila, Barbara Carrieri

**Affiliations:** 1 Department of Medical Science, University of Ferrara, Via Aldo Moro, 9, 44124 Ferrara, Italy; 2 Department of Translational Medical Sciences, University of Naples Federico II, Naples, Italy; 3 School of Medicine and Surgery, University of Milano-Bicocca, Milan, and Acute Geriatric Unit, San Gerardo Hospital, Monza, Italy; 4 Section of Geriatrics, Department of Medical Sciences, University of Turin, Città della Salute e della Scienza-Molinette, Torino, Italy; 5 Geriatria, Accettazione Geriatrica e Centro di Ricerca per l’Invecchiamento, , IRCCS-INRCA, Ancona, Italy; 6 Department of Clinical and Experimental Medicine, University of Messina, Messina, Italy; 7 Department of Experimental and Clinical Medicine, Research Unit of Medicine of Aging, University of Florence, Florence, Italy; 8 Geriatric Intensive Care Unit, Department of Geriatrics and Medicine, Azienda Ospedaliero-Universitaria Careggi, Florence, Italy; 9 Department Medicine and Surgery, Geriatric Rehabilitation Department, University of Parma, Parma, Italy; 10 Department of Advanced Medical and Surgical Sciences, University of Campania Luigi Vanvitelli, Naples, Italy; 11 Department of Medicine, Geriatrics Division, University of Verona, Verona, Italy; 12 Department of Geriatrics, Neurosciences and Orthopaedics, Catholic University of the Sacred Heart, Rome, Italy; 13 Center for Clinical Epidemiology, School of Medicine, University of Ferrara, Ferrara, Italy; 14 Department of Biomedical and Neuromotor Sciences, University of Bologna, Bologna, Italy

**Keywords:** Sarcopenic obesity, Sarcopenia, Diabetes, Acute care, Mortality

## Abstract

**Background:**

Although the prevalence of sarcopenic obesity is increasing, nowadays a universally accepted definition still does not exist. Because, this clinical entity is defined as the combination of obesity and sarcopenia, the diagnosis appears to be strictly linked to criteria used for sarcopenia and the available prevalence data are not uniform. To investigate the prevalence of sarcopenic obesity in older persons according to EWGSOP2 and FNIH criteria. Second, to evaluate the prevalence of diabetes in patients with sarcopenia diagnosed by the two definitions.

**Methods:**

Observational multicenter study performed in 2014 on older patients admitted to 12 Italian hospitals (GLISTEN Study). Data were collected through standardized questionnaires, which assessed: socio-demographic data, cognitive status, functional abilities, pharmacological therapy, comorbidities, and blood tests. Moreover, muscle mass and strength and physical performance were evaluated.

**Results:**

Six hundred and ten were included in the analyses. Among sarcopenic patients, the prevalence of sarcopenic obesity was 30.8% with FNIH and 0% with EWGSOP2 criteria. According to EWGSOP2 criteria, 23.7% of sarcopenic and 30.8% of non-sarcopenic patients were affected by diabetes (*p* = 0.101); otherwise, using FNIH criteria, 36.3% of sarcopenic and 26.9% of non-sarcopenic patients were diabetic (*p* = 0.030). After adjustment for potential confounders, diabetic patients had a 73% higher probability of being sarcopenic according to FNIH criteria (OR 1.73; 95% CI 1.13–2.64).

**Conclusions:**

The EWGSOP2 and FNIH sarcopenia criteria are differently related to the prevalence of obesity and diabetes. The EWGSOP2 criteria seem to be not suitable to identify people with sarcopenic obesity.

## Introduction

Prevalence of sarcopenic obesity is increasing worldwide, but estimates are not consistent across studies [1, 2]. There is not a diagnostic definition of sarcopenic obesity universally accepted and this condition is currently qualified as the co-occurrence of sarcopenia and obesity (i.e., Body Mass Index ≥ 30 Kg/m^2^) [3]: thus, the anthropometric and metabolic characteristics of patients with sarcopenic obesity might be substantially different according to the diagnostic criteria used for sarcopenia.

Sarcopenia is defined as an age-related decline of skeletal muscle mass with a consequent reduction in muscle strength and physical performance [4, 5]. The definitions currently used by the scientific community are those proposed by the Working Group on Sarcopenia in Older People (EWGSOP) in 2010 and revised in 2018—EWGSOP2 [6, 7] and by the Foundation for the National Institutes of Health (FNIH) Sarcopenia Project [8], that propose different and alternative methods for defining low skeletal muscle mass [9]. Both operational definitions suggest as cut-point for low muscle mass definition an appendicular lean mass (ALM) lower than 20 and 15 kg for men and women, respectively. Nevertheless, since muscle mass is correlated with body size, ALM can be adjusted for body size in different ways, i.e. using height squared (ALM/height^2^), weight (ALM/weight) or body mass index (ALM /BMI). EWGSOP2 consensus, recommends using ALM/height^2^ < 7.0 and < 5.5 kg/m^2^ in men and women, respectively, as cut-off points for low muscle mass definition [7] and FNIH recommendations include an ALM to BMI ratio (ALM/BMI) < 0.789 in men and < 0.512 in women [8].

We hypothesized that different methods for muscle mass standardization might capture different aspects of the sarcopenic phenotype with opposite anthropometric and metabolic characteristics, including but not limited to obesity and type 2 diabetes [10, 11], according to the diagnostic criteria used.

The aim of our study was, therefore, to investigate the prevalence of obesity and type 2 diabetes in older persons with sarcopenia defined according to different methods for muscle mass standardization, evaluating the concordance between the two methods.

## Methods

### Study design and data collection

Data were obtained from the Gruppo di Lavoro Italiano Sarcopenia—Trattamento E Nutrizione (GLISTEN) project, a cohort study performed in Geriatrics and Internal Medicine acute care wards of 12 Italian hospitals (Monza, Turin, Ferrara, Verona, Parma, Florence, Ancona, Rome, Napoli I, Napoli II, Cagliari, Messina). Methodology of the GLISTEN project has been described in detail elsewhere [12]. Briefly, the study was designed to investigate the prevalence and clinical correlates of sarcopenia in older hospitalized patients in Italy and to estimate the incidence of sarcopenia during hospital stay. All study centers obtained ethical approval from their institutions; participants signed a written informed consent. All patients consecutively admitted to the participating wards from February 2014 and May 2014 were screened for enrollment. Exclusion criteria were age younger than 65 years and patient’s unwillingness to take part in the study. All patients were assessed within 2 days since hospital admission and were followed until discharge. Participants’ data were collected through a standardized dedicated questionnaire including demographic characteristics, self-report functional status, cognitive, and mood assessment; medication use; incident and prevalent medical conditions; and biochemical test results. Objective measures of muscle mass (bioimpedance analysis [BIA]) and physical performance (handgrip strength and 4-m usual walking speed) were obtained at hospital admission and before discharge. In this study, 45 of the original 655 enrollees were excluded because of some baseline missing data, leading to a final sample of 610 persons (mean age 80.7 ± 6.6 years, male 48.7%).

### Assessment of sarcopenic obesity

Sarcopenic obesity was defined as the simultaneous presence of sarcopenia and a BMI ≥ 30 kg/m^2^ [13]. Sarcopenia was defined as the presence of low muscle mass and low muscle strength, according to EWGSOP2 [7] and FNIH criteria [8].

Muscle mass was measured by BIA using a Quantum/S Bioelectrical Body Composition Analyzer (Akern Srl, Florence, Italy). Whole-body BIA measurements were taken between the right wrist and ankle with the subject in a supine position, whenever possible. Appendicular Skeletal muscle Mass (ASM), equivalent to ALM, was calculated using the following equation by Sergi and colleagues: ASM (Kg) = − 3.964 + (0.227 × height^2^/resistance) + (0.095 × weight) + (1.384 × sex) + (0.064 × reactance) where height is measured in centimeters, resistance and reactance in ohms, weight in kilograms; for gender, men = 1 and women = 0. ASM was standardized by height squared (ASM/height^2^) and Body Mass Index (ASM/BMI) as suggested by EWGSOP2 and FNIH criteria. Low appendicular muscle mass was classified as ASM/height^2^ less than 7.0 kg/m^2^ in men and 6.0 kg/m^2^ in women, in line with EWGSOP2 cut-off points [14], and as an ASM/BMI ratio lower than 0.789 in men and 0.512 and women, according to FNIH [15].

Muscle strength was assessed by grip strength (GS), measured using a hand-held dynamometer (JAMAR hand dynamometer Model BK-7498, Fred Sammons Inc., Brookfield, IL). Three trials for each hand were performed, and the highest value of the strongest hand was used in the analyses. GS values below 27 kg in men and 16 kg in women were considered as abnormal according to the EWGSOP2 consensus [16]. The corresponding cut-offs for the FNIH criteria were 26 and 16 kg for men and women, respectively [17].

### Assessment of diabetes

Prevalent diabetes mellitus was defined as self-report of physician diagnosis or antidiabetic medication use. Among undiagnosed diabetic participants, presence of diabetes was identified by fasting plasma glucose level ≥126 mg/dL, based on the American Diabetes Association 2003 criteria. Current use of antidiabetic medications (oral antidiabetic agents and insulin) was determined during the baseline interview.

### Covariates

At hospital admission, a clinical interview was performed to every patient collecting sociodemographic variables, home pharmacological therapy and past medical history; Charlson Comorbidity Index (CCI) [18] was calculated to assess comorbidity burden. Functional status in basic activities of daily living (ADL) and cognitive status were evaluated as reported elsewhere [12]; specifically, difficulty in three or more activities was defined as severe ADL disability [19].

### Statistical analysis

Demographic and clinical features were presented using mean and standard deviation for continuous variables with approximately normal distribution or median and inter-quartile range [IQR] for numerical variables with asymmetric distribution; frequency and percentage were reported for dichotomous variables.

The characteristics of patients were compared according to gender and the presence of sarcopenia and sarcopenic obesity, using Student’s *t*-test and Pearson’s Chi-squared test for continuous and categorical variables, respectively.

The variables significatively related to the prevalence of sarcopenia were included in a multivariable logistic regression analysis and results were reported as odds ratio and 95% confidence interval (OR 95% CI). *P* value < 0.05 were considered statistically significant.

Statistical analyses were performed using Software R 3.5.0 (R Core Team (2020). R: A language and environment for statistical computing. R Foundation for Statistical Computing, Vienna, Austria. URL https://www.R-project.org/.).

## Results

Selected demographic and clinical characteristics of enrolled patients are shown in Table 1, according to the presence of sarcopenia. Participants with sarcopenia diagnosed by EWGSOP2 and FNIH definitions were 190 (29.5%) and 146 (23.9%), respectively. According to EWGSOP2 criteria, sarcopenic patients were older and had a lower BMI than participants without sarcopenia; moreover, the prevalence of osteoporosis, weight loss in the previous 6 months, and impaired cognitive function was more common in the sarcopenic subgroup. On the other hand, compared with patients without sarcopenia (FNIH criteria), a greater BMI and a higher prevalence of severe ADL disability, chronic obstructive pulmonary disease, type 2 diabetes, poorer cognitive performance, higher CCI and number of drugs was present among sarcopenic participants.

**Table 1:** Selected baseline characteristics according to different definitions

	EWGSOP2			FNIH		
	No Sarcopenia	Sarcopenia	*p*	No Sarcopenia	Sarcopenia	*p*
*N* (%)	471 (77.2)	139 (**22.8**)		464 (75.9)	146 (**23.9**)	
Age, mean ± SD	80.2 ± 6.5	82.4 ± 6.8	< 0.001	80.6 ± 6.6	80.9 ± 6.7	0.627
Male sex (%)	44.2	64.0	< 0.001	42.0	67.1	< 0.001
BMI, mean ± SD	**27.6 ± 4.9**	**22.5 ± 3.1**	** < 0.001**	**25.8 ± 4.8**	**28.2 ± 5.4**	** < 0.001**
Weight loss (%)	39.1	56.1	0.003	44.2	41.5	0.571
Severe ADL disability (%)	21.4	30.2	< 0.001	20.0	34.2	< 0.001
SPMSQ, median [IQR]	2 [1; 3]	2 [1; 4]	0.001	2 [1, 4]	3 [1, 4]	0.027
Congestive heart failure (%)	16.4	19.4	0.397	16.8	17.8	0.780
Type 2 diabetes (%)	**30.8**	**23.7**	**0.101**	**26.9**	**36.3**	**0.030**
COPD (%)	26.5	26.6	0.869	23.7	35.6	0.004
Charlson Comorbidity Index, median [IQR]	3 [2, 5]	3 [2, 4]	0.705	3 [1, 4	3 [2, 5]	0.027
Number of drugs, mean ± SD	6.1 ± 2.9	6.1 ± 2.7	0.837	5.9 ± 2.9	6.5 ± 2.7	0.450

*p* p value, *SD* standard deviation, *BMI* body mass index, *ADL* activity of daily living, *SPMSQ* Short Portable Mental Status Questionnaire, *IQR* interquartile range, *COPD* chronic obstructive pulmonary disease

**Fig. 1:**
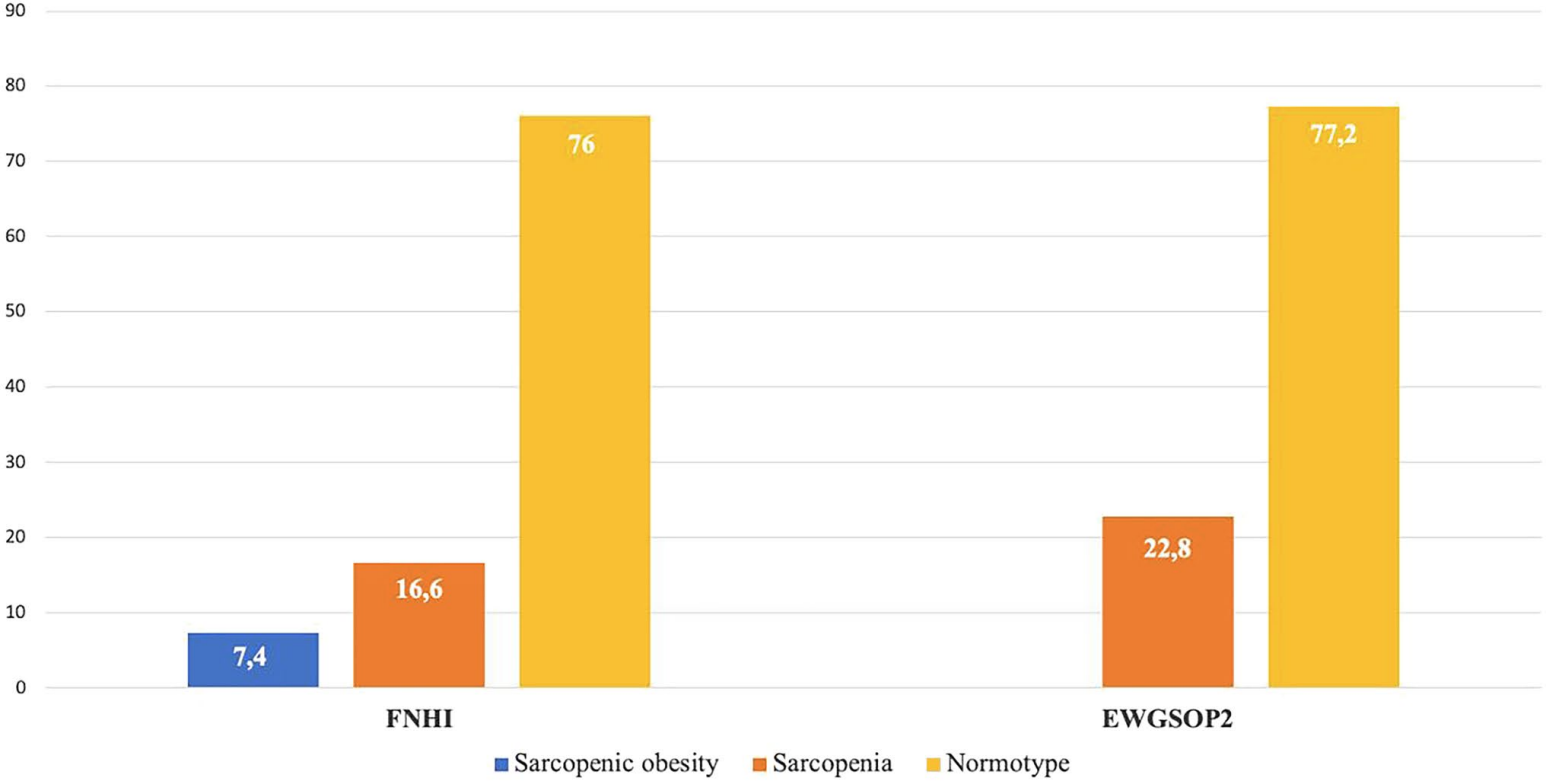
Prevalence of sarcopenic obesity according to FNIH and EWGSOP2 criteria

The prevalence of FNIH defined sarcopenic obesity was 7.4% (*n* = 45), whereas, no patients were defined as obese in the sarcopenic group based on EWGSOP2 criteria (Fig. 1). Moreover, according to FNIH definition, sarcopenic obesity was more common among women than among men (47.9% *vs* 22.4%, *p* = 0.003).

**Fig. 2:**
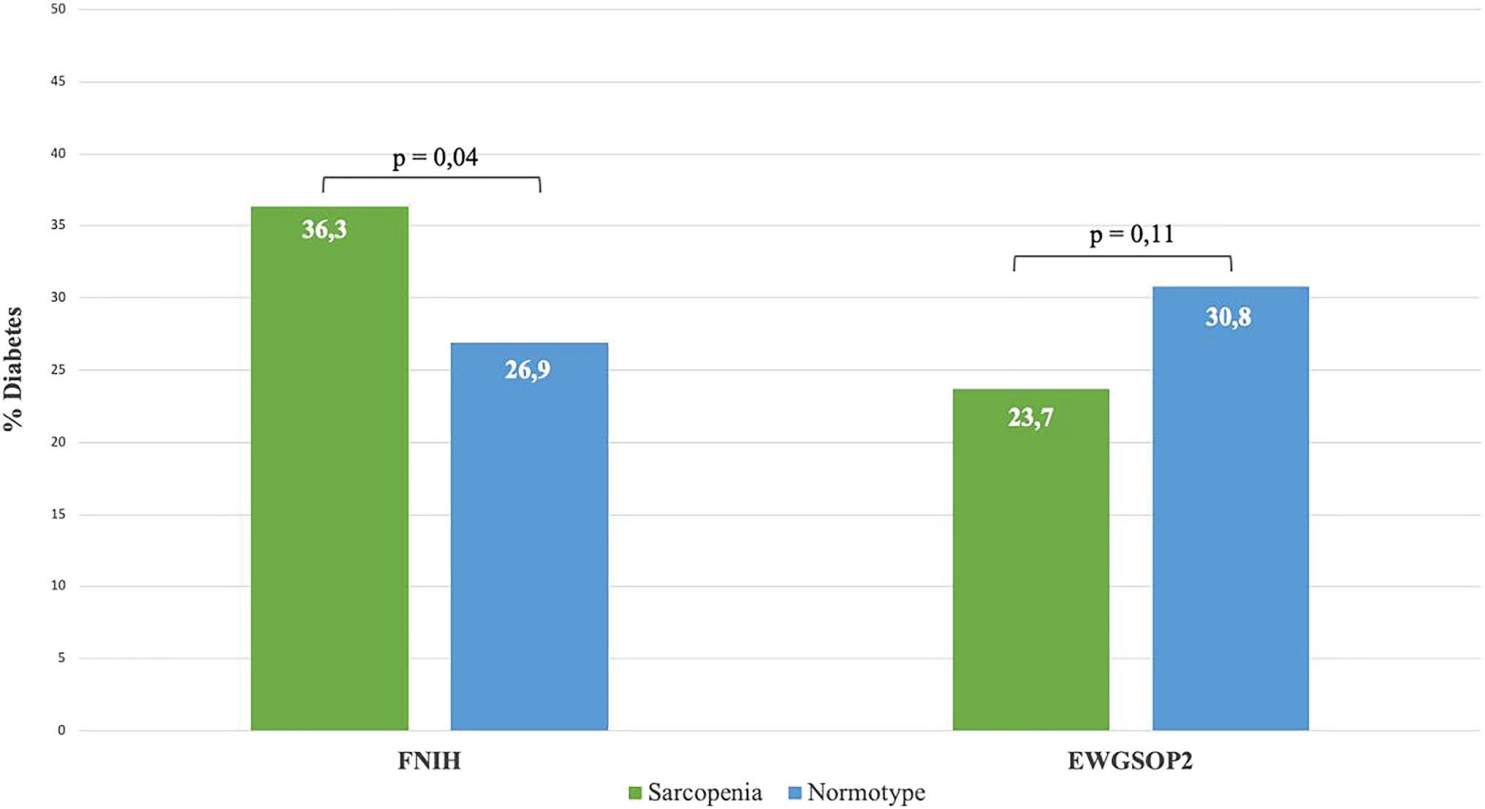
Prevalence of diabetes related to sarcopenia diagnosed by EWGSOP2 or FNIH criteria

Overall prevalence of diabetes was 32.8%, with likelihood of having diabetes being related to sarcopenia differently according to diagnostic criteria used. After muscle mass standardization for height^2^ (EWGSOP2 criteria), 23.7% of sarcopenic and 30.8% of non-sarcopenic patients were affected by diabetes (*p* = 0.101), whereas, after standardization by BMI (FNIH criteria), the prevalence of diabetes was 36.3% in sarcopenic subgroup and 26.9% in non-sarcopenic one (*p* = 0.030) (Fig. 2).

Finally, the strength of the association between the prevalence of diabetes and sarcopenia was assessed by multivariable logistic regression analysis.

In analyses adjusted for age and gender, patients with diabetes had a greater likelihood of being sarcopenic according to FNIH criteria (OR 1.73; 95% CI 1.13–2.64), whereas diabetes was not associated to sarcopenia prevalence when defined according to EWGSOP2 criteria (OR 0.84; 95% IC: 0.53–1.31). Adjustment for functional status and comorbidity did not modify the relationship between diabetes and sarcopenia (Table 2—Model 3). Additional adjustment for BMI (Table 2—Model 4) significantly attenuated the strength of the association between diabetes and FNIH-defined sarcopenia; conversely, the strength of the association with EWGSOP2 was substantially increased after adjustment for BMI, although without reaching the statistical significance (OR 1.44; 95% CI 0.91–2.27).

**Table 2:** Association between diabetes and sarcopenia by FNIH criteria definition adjusted for potential confounders

	Model 1 OR (95% CI)	Model 2 OR (95% CI)	Model 3 OR (95% CI)	Model 4 OR (95% CI)
FNHI criteria				
Diabetes	1.55 (1.04–2.29)	1.73 (1.13–2.64)	1.58 (1.04–2.41)	1.33 (0.87–2.04)
Age (years)	–	1.03 (0.68–1.57)	1.02 (0.67–1.54)	1.04 (0.68–1.58)
Gender (M)	–	2.94 (1.93–4.46)	3.16 (2.08–4.81)	3.88 (2.53–5.93)
BADL	–	–	1.17 (0.77–1.78)	1.16 (0.76–1.77)
SPMSQ	–	–	0.96 (0.63–1.46)	0.97 (0.63–1.48)
Charlson Index	–	–	1.05 (0.69–1.59)	1.02 (0.67–1.57)
BMI	–	–	–	1.13 (0.74–1.73)
EWGSOP2 criteria				
Diabetes	0.70 (0.45–1.08)	0.84 (0.53–1.31)	0.79 (0.50–1.25)	1.44 (0.91–2.27)
Age (years)	–	1.07 (0.68–1.67)	1.05 (0.67–1.67)	1.04 (0.66–1.65)
Gender (M)	–	2.65 (1.69–4.16)	2.73 (1.72–4.31)	4.73 (2.98–7.49)
BADL	–	–	1.10 (0.70–1.74)	1.17 (0.74–1.85)
SPMSQ	–	–	0.98 (0.62–1.54)	0.98 (0.62–1.55)
Charlson Index	–	–	1.02 (0.64–1.61)	1.09 (0.69–1.73)
BMI	–	–	–	0.68 (0.43–1.07)

*Model 1* unadjusted, *Model 2* adjusted for age and gender, *Model 3* adjusted for age, gender, Basic Activity of Daily Living, Short Portable Mental Status Questionnaire and Charlson Index, *Model 4* adjusted for age, gender, Basic Activity of Daily Living, Short Portable Mental Status Questionnaire, Charlson Index and Body Mass Index, *OR* odds ratio, *CI* confidence interval

## Discussion

Our data confirm the poor concordance between the two current diagnostic criteria of sarcopenia: indeed, the FNIH and the EWGSOP2 criteria identify different groups of patients and, particularly, the EWGSOP2 criteria do not capture people with sarcopenic obesity. Thus, we were able to identify patients with sarcopenic obesity only by using FNIH criteria, finding a prevalence of 7.4%. Because the methods and the cut-off used to evaluate muscle strength and physical performance are equivalent [7, 8], the different population identified by the two criteria are probably due to the different methods used to identify subjects with reduced muscle mass. Thus, while EWGSOP2 criteria standardizes the appendicular muscle mass for the patient’s height squared without considering the body weight, the FNIH criteria uses BMI, therefore, including the patient’s weight [7, 8]. Indeed, the two methods used for appendicular muscle mass standardization captured two groups of patients with significantly different BMI and different prevalence of obesity.

This is the first study that investigated the role of EWGSOP2 and FNIH criteria in estimating the prevalence of sarcopenic obesity in hospitalized older patients; our findings based on a multicenter and prospective cohort extended the results of previous reports [20, 21].

The prevalence of diabetes, a metabolic condition related to both obesity and sarcopenia, was also different in sarcopenic patients according to the two diagnostic criteria: using FNHI criteria, diabetes was significantly more common among the sarcopenic group than in the non-sarcopenic counterpart; conversely, patients with sarcopenia diagnosed by EWGSOP2 definitions had a lower prevalence of diabetes compared to non-sarcopenic patients [22, 23]. These results might be certainly explained by the different anthropometric characteristics of EWGSOP2-defined sarcopenic patients because these criteria tend to select underweight patients excluding those with obesity, a condition strongly associated with diabetes [24–26]. Moreover, several studies have demonstrated the presence of pathophysiological mechanisms linking diabetes to sarcopenia, including but not limited to the reduced muscle protein synthesis with increased protein catabolism caused by the direct effect of insulin resistance on muscle fibers or the reduction in moto-neurons due to diabetic peripherical neuropathy that leads to muscle atrophy [10, 27–29].

Likewise, in our study the association between diabetes and sarcopenia was confirmed also by multivariable logistic regression analysis, adjusting for potential confounders: diabetes was associated with a higher likelihood of having sarcopenia if assessed by FNIH criteria, while no correlation was demonstrated using EWGSOP2 criteria. Furthermore, in the final model adjusted for BMI, the association between diabetes and FNIH-defined sarcopenia was no longer significant reinforcing the role of obesity as a potential confounder. Using EWGSOP2 criteria, multivariable logistic regression analyses demonstrated an inverse relationship between diabetes and the likelihood of being sarcopenic; nevertheless after further adjustment for BMI the association became direct, with diabetic patients having almost a 50% increase likelihood of being sarcopenic, although the estimate did not reach the statistical significance. Overall, these finding support the hypothesis that body mass should be included into operational definitions of low muscle mass [30].

In interpreting these findings some limitations should be considered. First, the diagnosis of sarcopenic obesity was simply made considering the presence of sarcopenia in subjects with BMI ≥ 30. Second, the assessment of sarcopenia was performed in acutely ill patients, with possible transient impairment in muscle strength, unrelated to sarcopenia. Third, the study was conducted on a sample of hospitalized patient, thus these results have limited external validity.

## Conclusions

The method used for appendicular muscle mass standardization significantly affects the association of sarcopenia with obesity and type 2 diabetes, with standardization for height^2^ (EWGSOP2 criteria) having very low sensitivity for intercepting patients with sarcopenic obesity. Previous studies had already shown that EWGSOP2 and FNIH criteria have different construct and identify different patients: therefore, it is well known that they cannot be used interchangeably [20, 21, 31, 32]. Our findings add to this body of evidence, with a specific reference to the diagnosis of sarcopenic obesity and its relationship with diabetes. Further research is needed to examine consistency of sarcopenia definitions to overcome the discrepancy between current diagnostic criteria.

## Data Availability

The datasets of the current study are available from the corresponding author on reasonable request.

## Glisten study group investigators

Department of Medical Science, University of Ferrara, Ferrara, Italy: Gloria Brombo, Beatrice Ortolani, Elisabetta Savino, Elisa Maietti**. **Department of Clinical and Experimental Medicine, Geriatric Rehabilitation Department, University of Parma, Parma, Italy: Alberto Fisichella, Valeria Buttò. Department of Medicine, Section of Geriatrics, University of Verona, Verona, Italy: Mauro Zamboni, Cesare Caliari, Elena Ferrari. Research Unit of Medicine of Aging, Department of Experimental and Clinical Medicine, University of Florence, Florence, Italy. Geriatric Intensive Care Unit, Department of Geriatrics and Medicine, Azienda Ospedaliero-Univesitaria Careggi, Florence, Italy: Francesco Orso, Flavia Sacco, Maria Laura Di Meo. Department of Health Sciences, University of Milano Bicocca, Milano, Italy. Acute Geriatric Unit, S. Gerardo Hospital, Monza, Italy: Anna Paola Cerri, Marco Motta, Francesca Pittella, Alessandra Bonfanti. Department of Clinical and Experimental Medicine, University of Messina, Messina, Italy: Sergio Fusco, Roberto Schepisi, Christian Ferro, Antonino Catalano, Stefano Caruso, Luca Soraci. Dipartimento di Scienze Mediche, SCDU Geriatria e Malattie Metaboliche dell'Osso, Città della Salute e della Scienza, Molinette, Torino, Italy: Lorenzo Marchese, Luca Agosta. Department of Translational Medical Sciences, University of Naples Federico II, Naples, Italy: Claudia Basile. Dipartimento di Scienze Mediche, Chirurgiche, Neurologiche, Metaboliche e dell’Invecchiamento, Seconda Università di Napoli, Napoli, Italia: Carla Coppola, Anna Maria Dalise, Ilaria Fava. UOC di Geriatria Ospedale SS. Trinità ASL 8 Cagliari: Olga Catte, Maura Orru', Paolo Salaris. Department of Geriatrics, Neurosciences and Orthopaedics, Catholic University of the Sacred Heart, Rome, Italy: Anna Maria Martone, Elena Ortolani, Sara Salini. Geriatrics and Geriatrics Emergency Care, Italian National Research Center on Aging (IRCCS-INRCA), Ancona, Italy: Giuseppina dell’Aquila, Barbara Carrieri.
